# Language-Specific Differences in Large Language Model Diagnostic Reasoning: A Translation-Controlled Clinical Vignette Study

**DOI:** 10.3390/jcm15114082

**Published:** 2026-05-25

**Authors:** Jakub Magdziarz Ibrahim-El-Nur, Wojciech Kaczmarek, Weronika Winiarska, Adrian Kuś, Magdalena Łoś

**Affiliations:** 1 Department of Social Medicine and Public Health, Medical University of Warsaw, Pawińskiego 3a, 02-106 Warsaw, Poland; 2 Department of Non-Surgical Clinical Sciences, Faculty of Medicine, Wrocław University of Science and Technology, 50-370 Wrocław, Poland; 3 Centrum Medyczno-Diagnostyczne w Siedlcach, ul. Gen. F. Kleeberga 2, 08-110 Siedlce, Poland

**Keywords:** large language models, clinical decision support systems, natural language processing, differential diagnosis

## Abstract

**Background**: Large language models (LLMs) are increasingly being evaluated for clinically relevant diagnostic tasks, yet their performance may vary across languages. We aimed to determine whether input language influences LLM diagnostic reasoning in vignette-based clinical tasks and to inform multilingual predeployment evaluation for non-English healthcare systems. **Methods**: In this translation-controlled in silico study, 30 real-patient’s clinical vignettes were presented in paired English- and Polish-language conditions using back-translated prompts and cases. Six LLMs were evaluated with a structured reflection framework adapted from medical education. The study included 720 rater-level evaluations and 360 unique model–language–vignette responses. Responses were independently scored by 2 physician raters, with major discrepancies adjudicated by a third physician. The primary outcome was total rubric score. Secondary outcomes included differential diagnosis quality, justification, appropriateness of additional examinations, final diagnosis, and triage accuracy. Exploratory analyses assessed the number and cost of recommended examinations. **Results**: The effect of language differed significantly by model. Qwen2.5, Llama3.3, Meditron3, and OpenBioLLM performed significantly better in English, with the largest gap observed for Qwen2.5. GPT-5 and Bielik showed no statistically detectable English-Polish difference in overall score in this sample. Language-related differences were most evident in differential diagnosis quality, justification, and examination planning rather than in final diagnosis alone. Exploratory economic analyses suggested model- and language-dependent differences in testing burden, with broader suggested workups generally associated with higher diagnostic costs. Language robustness was not a consistent property of clinically evaluated LLMs. Performance differences were concentrated in reasoning and workup domains that are safety-relevant if these systems are used clinically. **Conclusions**: Multilingual clinical performance of LLMs is strongly model dependent. Language-specific evaluation should be considered before deployment in non-English healthcare systems.

## 1. Introduction

Diagnostic errors pose a substantial risk to patient safety and generate a significant financial burden on both patients and healthcare systems [1,2,3,4]. To address these issues, the integration of advanced technologies such as large language models (LLMs) is being actively investigated. These models are widely tested in medicine and have multiple applications, including treatment optimization, triage, and serving as AI-based Clinical Decision Support Systems (AI-CDSS) for diagnostic suggestions [5,6,7]. As LLMs are considered for integration into clinical workflows, their evaluation should account not only for overall performance but also for the language context in which they are expected to operate. While LLMs operate in many languages, they do not perform equally well in all of them [8,9,10,11]. In medicine, where even subtle degradations in reasoning quality may translate into unsafe triage, inappropriate diagnostic workups, or delayed recognition of serious conditions, language-dependent variability becomes a patient-safety concern rather than a purely technical limitation. This is particularly relevant for non-English healthcare systems, where local-language interaction is central to documentation, communication, and clinical decision-making, and where English-based validation may not adequately reflect real-world use. In Poland, AI is still rarely used, as few facilities currently utilize it [12]. While a limited number of early studies have attempted to quantify the language gap between Polish and English, primarily using multiple-choice question (MCQ)-based medical examinations [13,14] the Polish language is still largely underexplored in the broader context of LLM. Although multilingual differences in LLM performance have been reported previously, most existing studies have relied on MCQ-based benchmarks rather than clinically oriented reasoning tasks. In contrast, our study applies a structured reflection framework to assess differential diagnosis generation, justification, and workup planning, which more closely reflect potential clinical use. In addition to diagnostic reasoning quality, economic impact and test burden also warrant consideration, such evaluation may be relevant for understanding whether language-related performance differences are accompanied by differences in suggested diagnostic workup and testing burden. To our knowledge, this is also the first evaluation of the Polish LLM Bielik in a medical diagnostic reasoning setting and the first use of this methodology for Polish-language assessment [15,16]. This makes Polish a clinically meaningful test case for multilingual evaluation in a real-world non-English health system and highlights the need for predeployment assessment frameworks that can determine whether performance demonstrated in English generalizes safely to local-language clinical settings.

## 2. Materials and Methods

### 2.1. Objective

This study aimed to evaluate whether input language influences the diagnostic reasoning performance of different LLMs in clinically relevant vignette-based tasks. Using a structured reflection methodology derived from approaches employed to assess diagnostic reasoning in medical education, we compared paired Polish- and English-language task conditions, based on back-translated prompts and clinical vignettes, across multiple clinically relevant domains, including response relevance, quality of differential diagnosis, justification, triage accuracy, appropriateness of proposed investigations, and overall diagnostic accuracy. We also performed an exploratory assessment of potential differences in the resource burden associated with model-recommended workups. The study was designed to inform multilingual predeployment evaluation of LLMs for use in non-English healthcare systems.

### 2.2. Study Design

The study was accepted by the Medical University of Warsaw Bioethical Commission (approval number: AKBE/43/2026). This was a vignette-based in silico study that included an analysis of the impact of query language (Polish versus English). The assessment method was based on the structured reflection methodology used to evaluate diagnostic reasoning [17]. While LLMs benchmarks, including those in Poland, usually rely on datasets of single-choice questions from medical licensing exams [18,19,20,21] which are useful but often fail to adequately assess diagnostic reasoning. This study utilized a benchmark based on structured reflection. This methodology is traditionally used to assess clinical reasoning in medical students and is considered superior to standard multiple-choice testing for this purpose [22,23]. Similar methods for assessing LLMs have already been utilized in several studies [24,25]. However, to date, no studies have evaluated language models using this method within a Polish setting.

### 2.3. Assessment Method

**Figure 1:**
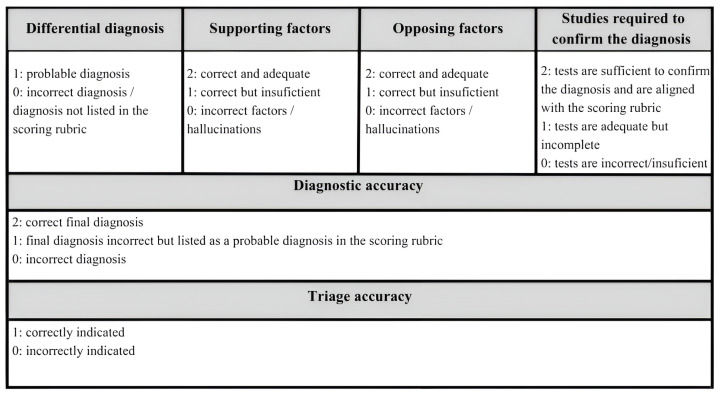
Scoring sheet. A model could score between 0 and 24 points. The model had to list three possible diagnoses. Because of this, the sections for differential diagnosis, supporting and opposing factors, and required studies were scored three times for each case. Diagnostic accuracy and the need for hospitalization (triage) were scored only once per case.

In this workflow, two independent physician raters assessed the responses from the language models, after which an experienced physician with over ten years of experience assessed discrepancies. Each model produced one response per vignette per language using a standardized prompt. These responses were independently graded by 2 expert raters, yielding 720 rater-level evaluations and 360 unique model–language–vignette responses. Interrater reliability was calculated between the two primary raters. Responses with marked disagreement were flagged for adjudication by a third physician rater. In total, 18 of 360 responses required adjudication, and for these cases the third-rater score replaced the mean of the two primary ratings in the final analytic dataset. The scoring sheet was structured as follows: for each case, the model could score 1 point for choosing a probable diagnosis listed in the scoring rubrics’s pool of diagnoses. For supporting factors, the model could score 2 points for correct responses, 1 point for partially correct responses, and 0 points for incorrect responses; the scoring for factors against the diagnosis followed the same rules. For the proper selection of additional tests, the model could score 2 points if the examination selection was sufficient to confirm the diagnosis and aligned with the scoring rubric, 1 point if the tests were adequate but incomplete, and 0 points if the tests were incorrect. Each model was asked to propose its three most probable diagnoses. A diagnostic accuracy rubric was also assessed: the model could score 2 points if the diagnosis was correct, 1 point if the diagnosis was incorrect but was listed by the model as a possible differential, and 0 points if the model chose an incorrect diagnosis outside the differential pool. Additionally, the model could score 1 point if it correctly indicated a hospitalization requirement (triage), and 0 if not. In total, a model could receive a maximum of 24 points. Scoring system is summarized in Figure 1.

### 2.4. Clinical Vignettes

A set of 40 clinical vignettes based on real-life patients was initially developed. An additional 14 vignettes were provided by the researchers of a landmark study by Goh et al. [24] which aimed to assess the effect of a LLM on physicians’ diagnostic reasoning, bringing the total to 54 considered vignettes. These cases were standardized and arranged into specific sections: History of Present Illness, Past Medical History, and Physical Examination, Laboratory Results. This arrangement was derived from a landmark study standard for evaluating computer-based diagnostic systems [26]. To select the final benchmark set, we used a nominal group technique adapted for vignette-based benchmark development. Three physician authors first agreed on case selection criteria and then independently reviewed all 54 candidate vignettes, flagging cases considered overly simplistic or excessively rare/complex for the intended diagnostic reasoning task. The final set of 30 vignettes was selected by consensus, with the aim of preserving clinical heterogeneity These selected vignettes reflected broad fields a family doctor might encounter, including cardiology, pulmonology, gastroenterology, endocrinology, nephrology, infectious diseases, neurology, and dermatology. To ensure these cases had never been included in any LLM training datasets, the vignettes were never publicly released. Finally, the vignettes were professionally back-translated, resulting in the development of two final sets in both the Polish and English languages. An example clinical vignette is provided in Appendix A.1.

### 2.5. Scoring Rubric

For each vignette from the set, a corresponding scoring rubric was developed. These scoring rubrics were initially created by two physician authors and subsequently verified by a board certified family physician with over ten years of experience. Each scoring rubric contained a pool of the most probable differential diagnoses, factors supporting and arguing against each diagnosis, a list of necessary additional examinations or studies required to confirm each diagnosis, and the estimated cost of those required examinations. Furthermore, the scoring rubric included the final definitive diagnosis for the case and a triage rubric stating whether hospitalization was required in that particular instance. An example scoring rubric is provided in Appendix A.1, Table A1.

### 2.6. Cost Analysis

Examination prices were obtained from the December 2025 price lists for uninsured patients at two university hospitals in Warsaw: the clinical Center of the Medical University of Warsaw (CCMUW) and Mazovian Bródnowski Hospital [27,28]. When tests were available at both institutions, CCMUW prices were prioritized. Prices for several missing tests were supplemented using December 2025 price lists from private facilities and the Military Medical Institute [29].

Exploratory economic analyses were performed at two levels. First, for diagnostically plausible entries accompanied by a perfect score for the “Additional tests necessary to confirm the diagnosis” rubric, we summed the cost of the examinations proposed by the model and calculated excess cost above the minimum required workup. This restriction was applied because incomplete or insufficiently specified workups may appear artificially less costly due to omission of relevant examinations. Second, to capture broader testing burden, we also summarized suggested diagnostic cost and number of suggested examinations across all diagnoses proposed by each model, regardless of diagnostic correctness.

### 2.7. Model Selection

In order to obtain a broad comparison, six language models were selected for the study. The chosen models represented the latest available versions as of January 2026. These specific models had been previously assessed for accuracy and the Polish versus English language gap by researchers from Poland [30] using sets of single-choice questions from the Polish Medical Final Examination (LEK) and the State Specialization Exam (PES). The models were divided into the following categories: Medical Models: OpenBioLLM-70B [31] OpenMeditron/Meditron3-70B [32]. General-Purpose Multilingual Models: Qwen 2.5 Instruct 72B version [33] Llama-3.3-70B-Instruct [34]. Polish-Specific Models: Bielik-11B-v2.2 [35]. Restricted API Models: GPT-5 [36]. To minimize the stochastic nature of LLMs and ensure high reproducibility of the clinical reasoning outputs, the generation temperature was set to 0 when this setting was available, except for GPT-5, for which the temperature parameter was not user-adjustable, and Llama3.3, for which a temperature of 0.3 was used because lower settings did not consistently yield coherent responses. The use of temperature 0 was also consistent with developer guidance for medical LLMs, such as OpenBioLLM, where deviations from recommended generation settings may be associated with degraded performance. Because these two models were evaluated under non-zero or non-user-controllable stochastic generation settings, GPT-5 and Llama3.3 were additionally evaluated in a second run to assess response variability across repeated generations. Finally, to allow for the use of non-quantized versions, the models were run on the Eagle Cluster at the Poznan Supercomputing and Networking Center [37]. GPT-5 was accessed via API; however, according to the available platform documentation, our workflow did not involve automatic translation of input prompts or model outputs. The remaining models were run directly on our own infrastructure, without any built-in translation layer.

### 2.8. Prompt Design

The prompt was standardized and subsequently back-translated, with the Polish prompt applied to the Polish cases and the English version applied to the English cases. The design followed prompt engineering rules for medical purposes [38] and consisted of a modified version of the prompt utilized in the landmark study by Goh et al. [24]. The Polish and English versions of the prompt are provided in Appendix A.2.

### 2.9. Statistical Analysis

All analyses were performed at the response level, with each observation representing one model response to one vignette in one language.

The primary outcome was the total rubric score. Secondary outcomes included rubric subdomain scores for plausible differential diagnoses, supporting factors, opposing factors, additional examinations, final diagnosis, and triage classification. Additional analyses included exploratory economic outcomes related to the number and cost of suggested additional examinations.

Interrater reliability between the 2 primary raters was assessed before adjudication. For the continuous total score, intraclass correlation coefficients (ICC) were calculated using a 2-way random-effects model for absolute agreement, with both single-rater and mean-of-2-raters estimates reported. For ordinal final diagnosis scoring, weighted Cohen’s *κ* with squared weights was calculated. For binary triage classification, Cohen’s *κ* was calculated. Internal consistency of the composite rubric was summarized using Cronbach’s α. Bland–Altman analysis was used as a supplementary measure of agreement for the total score.

To assess the primary study question of language bias, linear mixed-effects models were fitted with total score as the dependent variable, fixed effects for language, model, and their interaction, and a random intercept for vignette to account for repeated evaluation of the same case across models and languages. The primary hypothesis test was the language-by-model interaction, assessed by likelihood-ratio comparison of models with and without the interaction term. Model-specific English-minus-Polish contrasts were then estimated from the interaction model using estimated marginal means. Because these were prespecified pairwise comparisons within the primary outcome family, Holm correction was applied across the 6 model-specific contrasts.

Continuous secondary rubric outcomes were summarized descriptively by model. For binary paired outcomes, including strict final diagnosis correctness and triage accuracy, within-model English-versus-Polish comparisons were evaluated using exact McNemar tests.

Economic variables, including the number of suggested examinations, total cost of suggested examinations, minimal cost required by the scoring rubric, and excess cost above the minimal workup, were analyzed separately from rubric outcomes and were considered exploratory. They were summarized descriptively.

Assumptions of the primary mixed-effects model were assessed using residual-versus-fitted plots, Q–Q plots of residuals and random effects, and formal checks for singular fit, collinearity, and heteroscedasticity. Independence between vignettes and independence of raters were considered design assumptions. Heteroscedasticity was assessed using the Breusch–Pagan test for residuals versus fitted values and the Fligner–Killeen test for residual spread across model-language groups. Collinearity diagnostics were examined, recognizing that elevated variance inflation factors can occur in fully interacted categorical models. All tests were 2-sided, and statistical significance for the primary analysis was defined as α=0.05. Secondary and exploratory analyses were interpreted with emphasis on effect sizes and 95% confidence intervals. Statistical analyses were performed in R, version 4.5.2 (R Foundation for Statistical Computing).

## 3. Results

**Figure 2:**
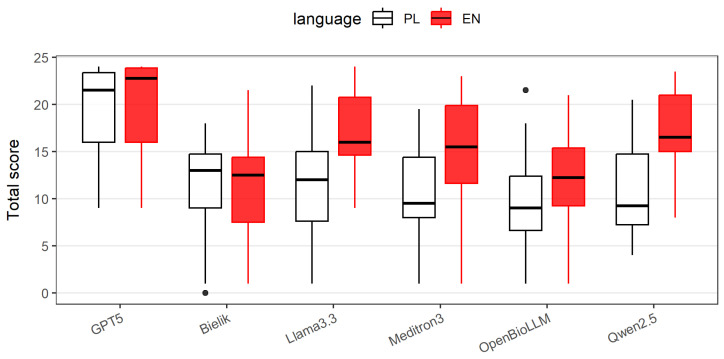
Distribution of total scores by model and prompt language.Boxplots show response-level total rubric scores across 30 clinical vignettes for each model in Polish (PL) and English (EN). Center lines indicate medians; boxes, interquartile ranges; whiskers, 1.5 times the interquartile range; and points, outliers.

Thirty translation-controlled clinical vignettes were evaluated in 2 languages (English and Polish) by 6 LLMs: GPT-5, Bielik, Llama3.3, Meditron3, OpenBioLLM, and Qwen2.5. Each model generated 1 response per vignette per language using a standardized prompt, yielding 360 unique model-language-vignette responses. Two expert raters independently scored all responses. For the main numeric analyses, response-level scores were defined as the mean, except for 18 flagged responses with marked disagreement, for which third rater served as adjudicator and his score was used. Overall performance is presented in Figure 2 and Table 1.

### 3.1. Interrater Reliability

Agreement between the 2 primary raters was high. For the primary endpoint (total score, 0–24), the intraclass correlation coefficient was 0.972 for a single rater and 0.986 for the mean of 2 raters. Agreement was also high for final diagnosis scoring (weighted Cohen *κ*, 0.931) and triage classification (Cohen *κ*, 0.980). Overall, 125 responses received differing scores between the 2 primary raters; however, most disagreements were small, typically differing by 1–2 points. To focus adjudication on marked discrepancies, we selected responses in the top 5% of maximum absolute score differences. This resulted in 18 responses being adjudicated, with absolute score differences ranging from 3 to 8 points. Adjudication was therefore limited to responses with substantial disagreement, and we did not observe evidence that it had a systematic effect on the overall pattern of results. Supplementary internal consistency of the composite rubric was acceptable (Cronbach α=0.71), supporting use of the total score as a summary measure while recognizing that the rubric captures related but distinct domains.

### 3.2. Primary Outcome: Overall Performance by Language

In a mixed-effects model of total score including fixed effects for model, language, and their interaction, with a random intercept for vignette, the estimated effect of language differed by model (likelihood-ratio test for language-by-model interaction: $\chi^{2}=33.0$; df = 5; p<0.001). Model diagnostics did not identify major violations of assumptions. Model-specific English-minus-Polish contrasts are presented as estimated mean differences with 95% confidence intervals to emphasize the magnitude and precision of the language effect.

GPT-5 showed no meaningful language difference in total score (mean English, 19.23; mean Polish, 19.25; difference, −0.02 points; 95% CI, −2.06 to 2.03; Holm-adjusted p>0.05). The estimate was centered near zero, and the confidence interval was compatible with little or no language effect in either direction. Bielik also showed no statistically detectable language difference in this sample (mean English, 11.42; mean Polish, 11.07; difference, 0.35 points; 95% CI, −1.70 to 2.40; Holm-adjusted p>0.05), with a small point estimate and a confidence interval that included no effect.

**Figure 3:**
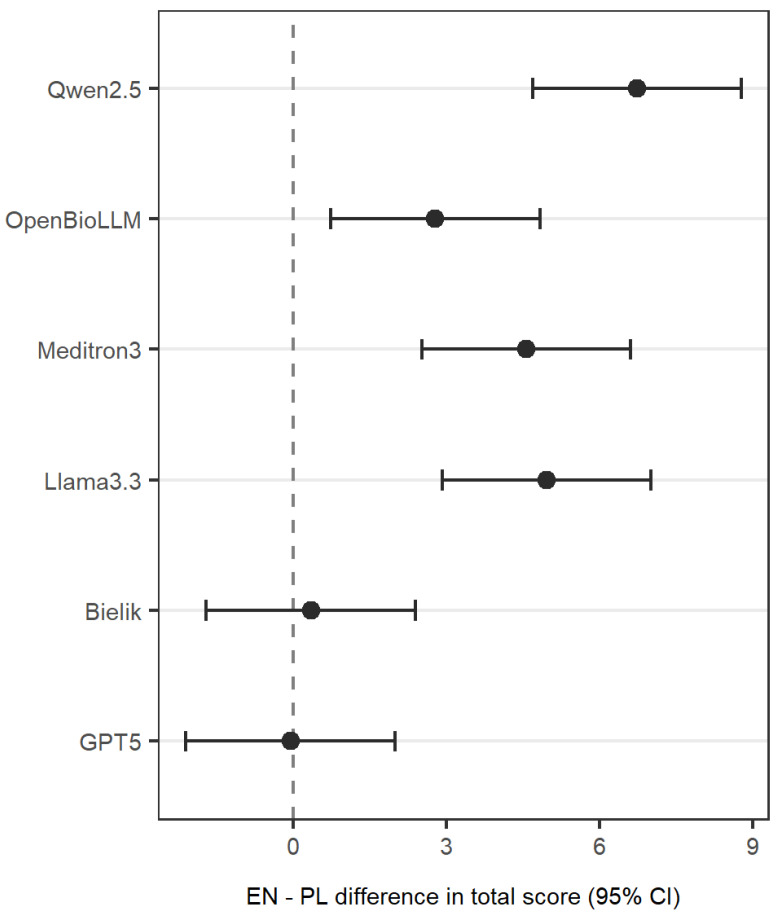
Estimated language effect on total score by model.Points indicate the estimated mean difference in total score between English and Polish prompts (EN minus PL) for each model, with horizontal error bars representing 95% confidence intervals from the linear mixed-effects model. Positive values indicate higher scores with English queries.

By contrast, several models had larger estimated English-minus-Polish differences in total score (Figure 3). The estimated English-minus-Polish difference was 6.73 points (95% CI, 4.69–8.78; Holm-adjusted p<0.001) for Qwen2.5, 4.97 points (95% CI, 2.92–7.01; Holm-adjusted p<0.001) for Llama3.3, 4.57 points (95% CI, 2.52–6.61; Holm-adjusted p<0.001) for Meditron3, and 2.78 points (95% CI, 0.74–4.83; Holm-adjusted p<0.01) for OpenBioLLM. On the 0–24 total-score scale, these differences corresponded to approximately 28.0%, 20.7%, 19.0%, and 11.6% of the maximum possible score, respectively. In practical terms, the 6.73-point English advantage observed for Qwen2.5 was comparable to nearly one complete differential-diagnosis entry on the rubric, including the suggested diagnosis, justification, and additional examinations. These findings suggest that language bias was model dependent rather than uniform across models, ranging from negligible estimated differences for GPT-5 and Bielik to larger English advantages for Qwen2.5, Llama3.3, Meditron3, and OpenBioLLM.

### 3.3. Differential Diagnosis Performance

Performance in generating plausible differential diagnoses differed by model and language. GPT-5 had the highest and most stable differential-diagnosis performance across languages (mean plausible differential score, 2.40 in English and 2.47 in Polish, suggesting little language-related difference in this domain. Qwen2.5 and Llama3.3 performed substantially better in English than in Polish, with mean plausible differential scores increasing from 1.53 to 2.33 for Qwen2.5 (difference, 0.80 points) and from 1.75 to 2.30 for Llama3.3 (difference, 0.55 points). For Qwen2.5, this difference corresponded to nearly one additional plausible differential diagnosis generated in English compared with Polish, on average. Bielik and OpenBioLLM generated fewer plausible differential diagnoses overall.

### 3.4. Final Diagnosis Performance

Strict final-diagnosis correctness, based on adjudicated consensus, varied by model. GPT-5 had the highest strict correctness in both languages (96.7% in English and 89.7% in Polish; English-minus-Polish difference, 7.0 percentage points), followed by Qwen2.5 (80.0% and 75.0%; difference, 5.0 percentage points) and Llama3.3 (79.3% and 70.4%; difference, 8.9 percentage points). Meditron3, OpenBioLLM, and Bielik had lower overall strict correctness. In paired within-model analyses using exact McNemar tests, no model-specific English–Polish difference in strict final-diagnosis correctness remained statistically significant after multiplicity correction. Given the limited power of these binary endpoint analyses, particularly for model-specific comparisons, these results should be interpreted cautiously and alongside the observed absolute percentage-point differences.

### 3.5. Triage Classification

Triage classification accuracy was generally high but varied by model. GPT-5 achieved 93.1% accuracy in English and 86.7% in Polish (English-minus-Polish difference, 6.4 percentage points). Qwen2.5 achieved 93.3% accuracy in English and 76.7% in Polish (difference, 16.6 percentage points), and Llama3.3 achieved 93.1% and 75.9%, respectively (difference, 17.2 percentage points). Bielik, Meditron3, and OpenBioLLM showed intermediate performance. Within-model paired analyses did not detect statistically significant language differences after multiplicity adjustment. Given the limited power of these binary endpoint analyses, particularly for model-specific comparisons, these results should be interpreted cautiously and alongside the observed absolute percentage-point differences.

### 3.6. Exploratory Economic Outcomes

The exploratory economic analysis suggested model- and language-dependent differences in the resource intensity of suggested diagnostic workups (Table 2). Cost outcomes were calculated per plausible differential diagnosis that was accompanied by a perfect score for additional examinations. This restriction was applied because incomplete or insufficiently specified workups may appear artificially less costly due to omission of relevant examinations. Therefore, the suggested cost, excess cost, and overtesting estimates should be interpreted as describing the resource intensity of selected diagnostically plausible and sufficiently complete workups, rather than the overall cost of all model outputs.

Among eligible diagnostic entries, GPT-5 generated the highest suggested diagnostic costs in both languages, with median suggested costs of 1201 PLN in Polish and 1317 PLN in English. GPT-5 also showed high excess costs above the minimum required workup, with overtesting observed in 100.0% of eligible entries in both languages. These findings suggest that, among sufficiently complete diagnostic entries, GPT-5 tended to propose more extensive diagnostic testing. Qwen2.5 also showed relatively high suggested costs and frequent overtesting, with median suggested costs of 731 PLN in Polish and 722 PLN in English and overtesting rates of 100.0% and 96.7%, respectively.

In contrast, OpenBioLLM appeared to generate lower-cost workups among eligible diagnoses, particularly in English, with median suggested costs of 513 PLN in Polish and 340 PLN in English. It also had the lowest excess-cost estimates, with median excess costs of 34 PLN in Polish and 0 PLN in English, and lower observed overtesting rates, especially in English. However, these lower costs should be interpreted cautiously, because more economical workups may reflect either more selective test choice or differences in the set of entries that met eligibility criteria.

The association between prompt language and diagnostic resource use did not appear uniform across models. Bielik and Llama3.3 showed higher suggested costs and higher excess costs in English than in Polish, suggesting that English prompting may have been associated with more extensive workups for these models. Conversely, Meditron3, OpenBioLLM, and Qwen2.5 showed comparable or lower excess costs in English among eligible entries. GPT-5 remained relatively high-cost in both languages, without a clear consistent cost reduction in either direction. Overall, these exploratory findings suggest that prompt language may influence not only diagnostic reasoning performance, but also the resource intensity of model-generated recommendations. Because the economic analyses were exploratory and restricted to selected eligible diagnostic entries, these results should be interpreted descriptively and as hypothesis-generating, with emphasis on the magnitude and direction of model- and language-specific differences rather than formal inference.

To complement the filtered excess-cost analysis, we also examined suggested testing burden across all diagnoses proposed by each model, without restricting the analysis to plausible diagnoses or complete examination-score categories. This broader analysis showed substantial between-model differences in both the number of suggested examinations and the overall suggested diagnostic cost (Table 3). GPT-5 generated the highest suggested testing burden in both languages, with median suggested costs of 3691 PLN in Polish and 3774 PLN in English, and approximately 19 examinations suggested per case. Qwen2.5 also generated relatively high suggested costs and examination counts, whereas OpenBioLLM produced the lowest suggested testing burden, particularly in English, with a median suggested cost of 687 PLN and a median of 3 suggested examinations. Bielik, Llama3.3, and Meditron3 occupied an intermediate range. Language-associated differences were model dependent rather than uniform: suggested costs were higher in English for Bielik and Llama3.3, lower in English for OpenBioLLM and Qwen2.5, and broadly comparable across languages for GPT-5 and Meditron3 based on paired EN–PL differences. Because this analysis includes all proposed diagnoses regardless of diagnostic plausibility or correctness, it should be interpreted cautiously as a secondary description of model-generated testing burden rather than a measure of clinically appropriate cost.

### 3.7. Repeat-Run Sensitivity Analysis

Because GPT-5 and Llama3.3 were evaluated under stochastic or non-user-controllable generation settings, we performed a second run for these models to assess repeat-run stability. Total-score summaries were broadly similar between runs for both models and both languages (Table 4). Although some run-to-run variation was observed, it remained limited relative to the main between-language patterns in the primary analysis.

## 4. Discussion

In this translation-controlled benchmark study of 6 large language models applied to clinical vignette reasoning in English and Polish, we found that the association between language and performance differed substantially across models. Some models showed little evidence of language sensitivity, whereas others performed materially better in English than in Polish. Importantly, the study was designed primarily to assess within-model differences between English and Polish under translation-controlled conditions, rather than to establish a direct performance ranking between smaller and larger models. 

The primary finding was a strong language-by-model interaction for the total score. GPT-5 showed similar overall performance in English and Polish, and notably, Bielik also did not demonstrate the Polish-language advantage that might have been expected from a Polish-specific model, instead showing broadly similar performance across both languages. By contrast, Qwen2.5, Llama3.3, Meditron3, and OpenBioLLM showed higher total scores in English. These results suggest that language robustness should not be assumed to be a general property of medical LLM performance. Instead, it appears to be model specific.

The repeat-run sensitivity analysis for GPT-5 and Llama3.3 was broadly consistent with this interpretation: repeated generations produced similar total-score summaries, and run-to-run differences did not materially change the overall pattern of the observed language effects.

An important feature of these findings is that language effects were more evident in reasoning and workup domains than in final-diagnosis labeling alone. This distinction may be relevant for the clinical evaluation of LLM-based decision-support tools. In potential clinical use, the value of an LLM is not limited to proposing a final diagnosis; it may also affect how clinicians frame the differential diagnosis, interpret supporting and opposing evidence, and choose further evaluation. A model that preserves label accuracy while providing weaker reasoning in a non-English language may still warrant more cautious evaluation for clinical decision-support use. This model-specific pattern is broadly consistent with the Polish-English medical exam benchmark reported by Grzybowski et al., in which Qwen2.5-72B, OpenBioLLM-70B, and Meditron3 generally performed better on English versions of Polish medical examinations, whereas Bielik showed a relative Polish advantage on some subsets and GPT-family models showed only small or inconsistent cross-lingual differences [30]. At the same time, our study extends those observations beyond answer selection accuracy by showing that the language effect persists in clinician-rated diagnostic reasoning, not only in exam-question performance. Most prior multilingual evaluations of medical LLMs, including the Polish–English benchmark by Grzybowski et al. and recent language-specific studies in Swedish and Japanese, have relied primarily on exam-style or benchmark-based testing; in contrast, our study applies a structured reflection framework to clinician-rated diagnostic reasoning. Our interpretation is also consistent with broader cross-lingual healthcare LLM research suggesting that English often remains the strongest operating language in medical tasks [39] as well as with recent language-specific benchmark efforts in Swedish and Japanese, which similarly underscore the need for local-language validation in non-English medical contexts [40,41].

Triage classification accuracy was encouraging for several models, particularly in English, although the observed misclassification rates indicate that further validation would be needed before any higher-stakes clinical use. The study was also limited in its ability to evaluate rare but high-consequence triage errors. These findings may be more relevant to clinician-support scenarios than to independent triage applications.

The exploratory economic analyses suggest that language-related differences in performance may also be accompanied by variation in the intensity of suggested diagnostic workup. In the filtered analysis, limited to diagnostically plausible and sufficiently complete workups, GPT-5 and Qwen2.5 tended to generate the highest suggested costs and the greatest excess above the minimum required workup, whereas OpenBioLLM generally produced lower-cost workups. The broader analysis across all diagnoses proposed by each model further indicated that these differences were not restricted to eligible diagnostically correct entries, but also reflected more general model-specific tendencies in suggested testing burden. Taken together, these findings may suggest that, for some models, language-related gains in rubric performance may be associated with greater diagnostic utilization. Nevertheless, the economic analyses were exploratory and based on benchmark-derived testing suggestions rather than observed clinical decisions or formal health-economic evaluation, and should therefore be interpreted cautiously. Further research should examine whether language-related differences in diagnostic reasoning are associated with systematic differences in testing burden and downstream resource use.

This study has several strengths. The translation-controlled vignette design reduced semantic drift and strengthened inference about language effects. The paired within-vignette comparison allowed direct English-Polish evaluation under matched clinical content. The rubric captured multiple clinically relevant domains beyond final diagnosis alone. In addition, dual expert scoring with targeted adjudication yielded high interrater reliability for the primary outcome and key secondary endpoints. Another strength of this study is that the clinical vignettes were based on real-world medical documentation, yielding unseen cases derived from authentic clinical record rather than publicly available benchmark datasets. A further strength is the use of structured reflection as the primary evaluative framework. Similar to prior work adapting structured reflection for LLM-related diagnostic reasoning research, this approach offers a richer and more clinically meaningful assessment of reasoning quality than benchmarks limited to final-answer accuracy [24].

This study also has limitations. The sample included 30 vignettes, which provided reasonable power for continuous-score comparisons but limited power for binary model-specific endpoints. Most models generated one response per vignette per language, so within-model variability across repeated generations could not be comprehensively assessed for all models. However, because GPT-5 and Llama3.3 were evaluated under stochastic or non-user-controllable generation settings, we performed an additional repeat run for these two models. The resulting total-score summaries were broadly similar between runs and did not alter the interpretation of the main findings. Nevertheless, larger future studies should assess repeated generations across all evaluated models and languages. This was partly a consequence of the structured reflection methodology, which required each response to be manually evaluated by physician raters rather than scored automatically as in MCQ-based benchmarks. We also used a single standardized prompt and low-temperature settings to maximize comparability across models and languages; however, this may limit generalizability to other prompting strategies and sampling conditions. Reducing the candidate set from 54 to 30 vignettes may also have introduced selection bias, and future studies should evaluate larger and more diverse case sets. In addition, because the vignettes were derived from non-public clinical materials and were intentionally not released to avoid contamination of future benchmarks, full external reproducibility is limited. Moreover the findings reflect the specific model versions examined here and should be interpreted within the constraints of this study design. The economic analysis was exploratory and based on recorded suggestions aggregated across diagnosis slots. Future studies should extend this framework to larger vignette sets, additional non-English languages. An important next step in the Polish setting would be prospective evaluation of physician–LLM interaction in simulated or real primary care workflows to examine whether language-related differences affect diagnostic decisions, resource use, and clinically-related outcomes.

## 5. Conclusions

These findings may have practical implications for further evaluation of multilingual clinical applications. For models with little evidence of language sensitivity, direct Polish prompting may warrant further evaluation. For models with substantial English advantage, translation-mediated workflows may improve performance and could be explored in further studies. Nevertheless such workflows introduce additional considerations, including privacy, latency, translation error, and potentially increased downstream testing. Accordingly, language-specific validation may be important when assessing LLMs for potential use in non-English settings.

In summary, language bias in clinical vignette reasoning was present in this study but was strongly model dependent. These findings support further model-specific and language-specific evaluations in larger studies before clinical implementation is considered in multilingual healthcare settings.

## Figures and Tables

**Table 1:** Descriptive Summary of Rubric Scores by Model and Language.

Model	Lang	Total Score	Plausible DDx	Justification	Exams Score	Diag. Acc. (%)	Triage Acc. (%)
GPT5	PL	21.5 [16.0, 23.0]	3.00 [2.00, 3.00]	10.50 [7.25, 12.00]	4.50 [4.00, 6.00]	89.7	86.7
	EN	22.8 [16.0, 23.9]	3.00 [2.00, 3.00]	11.00 [7.62, 12.00]	5.00 [4.00, 6.00]	96.7	93.1
Bielik	PL	13.0 [9.0, 14.8]	2.00 [1.00, 2.00]	4.75 [3.00, 6.50]	2.87 ± 1.66	46.4	83.3
	EN	11.4 ± 5.4	2.00 [1.00, 2.00]	4.53 ± 2.80	2.95 ± 1.78	56.7	80.0
Llama3.3	PL	12.0 ± 5.9	2.00 [1.00, 2.00]	5.30 ± 3.07	2.70 ± 1.72	70.4	75.9
	EN	17.0 ± 4.6	2.00 [2.00, 3.00]	8.12 ± 2.63	3.50 [2.62, 5.88]	79.3	90.0
Meditron3	PL	10.4 ± 4.9	2.00 [1.00, 2.00]	4.00 ± 2.41	2.48 ± 1.62	62.1	83.3
	EN	14.9 ± 5.5	2.00 [2.00, 3.00]	6.83 ± 3.12	3.60 ± 1.52	70.0	76.7
OpenBioLLM	PL	9.5 ± 4.9	1.50 [1.00, 2.00]	3.60 ± 2.41	2.27 ± 1.56	50.0	80.0
	EN	12.3 ± 5.3	2.00 [1.25, 2.00]	5.10 ± 2.74	3.05 ± 1.64	63.3	80.0
Qwen2.5	PL	9.2 [7.2, 14.8]	1.00 [1.00, 2.00]	4.25 ± 2.24	2.00 [2.00, 3.88]	75.0	76.7
	EN	17.4 ± 4.4	2.00 [2.00, 3.00]	8.08 ± 2.43	4.00 [3.00, 6.00]	80.0	93.3

Note: Numeric rubric scores are presented as Mean ± SD for normally distributed data, and Median [IQR] for non-normally distributed data, based on Shapiro-Wilk testing. Total Score is out of a maximum of 24 points. Plausible Differential Diagnosis (DDx) is out of 3. Justification (factors for and against summarized) is out of 12. Studies required to confirm diagnosis (Exams Score) is out of 6. Final diagnostic accuracy and triage accuracy reflect consensus accuracy across all 30 vignettes.

**Table 2:** Cost (PLN ) and examination-related metrics are shown per plausible differential diagnosis with a maximal score for additional examinations. EN–PL Diff represents the paired excess-cost difference between English and Polish outputs.

Model	Lang	*n*	Sugg. Cost	Excess Cost	Sugg. Exams	Overtest (%)	EN–PL Diff
GPT5	PL	66	1201 [961, 2103]	827 [568, 1626]	6.0 [5.0, 7.6]	100.0	− 52.5 [−333.0, 413.5]
	EN	66	1317 [944, 1951]	1216 ± 869	7.0 [5.1, 7.7]	100.0	
Bielik	PL	37	270 [194, 424]	167 ± 192	2.7 ± 1.0	91.7	77.0 [−59.5, 311.7]
	EN	39	524 [318, 808]	297 [99, 382]	3.0 [2.5, 3.0]	95.5	
Llama3.3	PL	32	412 [227, 548]	150 [55, 220]	2.3 [2.0, 3.0]	95.5	24.5 [0.0, 263.0]
	EN	53	462 [290, 989]	242 [70, 469]	2.8 [2.0, 3.0]	89.7	
Meditron3	PL	31	567 ± 325	193 [0, 497]	2.8 ± 1.2	71.4	−116.5 [−321.5, 186.0]
	EN	46	440 [204, 843]	121 [0, 438]	2.0 [1.5, 3.0]	70.4	
OpenBioLLM	PL	24	513 ± 349	34 [14, 225]	2.0 [2.0, 2.0]	76.5	−13.5 [−84.0, 4.8]
	EN	38	340 [184, 688]	0 [0, 116]	1.2 [1.0, 2.0]	45.8	
Qwen2.5	PL	37	731 [400, 1120]	374 [257, 606]	3.0 [3.0, 3.9]	100.0	−15.8 [−211.8, 416.9]
	EN	58	722 [310, 1204]	250 [121, 780]	3.0 [2.8, 3.9]	96.7	

Note: Cost (PLN) and exam metrics are calculated per plausible differential diagnosis (*n*) accompanied by perfect score for additional exams presented as Median [IQR] or Mean ± SD depending on normality. EN–PL Diff represents the paired excess cost difference (Median [IQR]) between languages.

**Table 3:** Suggested diagnostic cost and number of suggested examinations by model and language. Suggested cost (PLN) and suggested examinations are reported across all diagnoses proposed by each model, without restriction to plausible differential diagnoses or examination-score categories. EN–PL Cost Diff indicates the paired English-minus-Polish difference in suggested cost.

Model	Lang	Suggested Cost (PLN)	Suggested Exams	EN–PL Cost Diff
GPT5	PL	3691 [2892, 6664]	19.7 ± 6.0	−90.0 [−980.2, 875.5]
	EN	3774 [2680, 5853]	18.8 ± 5.7	
Bielik	PL	1557 [835, 2357]	8.5 [6.0, 10.0]	347.5 [−91.8, 930.2]
	EN	1806 [1389, 2518]	9.0 [7.0, 9.8]	
Llama3.3	PL	1391 [1066, 2077]	6.5 ± 1.9	317.5 [−50.0, 950.0]
	EN	1790 [981, 3378]	7.1 ± 2.3	
Meditron3	PL	1507 [881, 2599]	7.8 ± 3.5	−90.5 [−536.0, 459.0]
	EN	1786 [635, 1966]	6.5 [4.0, 7.8]	
OpenBioLLM	PL	1212 [842, 1705]	5.5 [4.0, 6.0]	−315.5 [−775.2, 139.2]
	EN	687 [396, 1681]	3.0 [3.0, 6.0]	
Qwen2.5	PL	2399 ± 1296	10.0 [9.0, 12.0]	−291.5 [−918.5, 332.5]
	EN	2272 [942, 3066]	9.5 ± 1.7	

Note: Suggested Cost (PLN) was calculated from all examinations natively suggested by the model. Suggested Exams are presented as Median [IQR] or Mean ± SD depending on distribution. EN–PL Cost Diff represents the paired English-minus-Polish difference in suggested cost, shown as Median [IQR].

**Table 4:** Comparison of Run 1 and Run 2 total rubric scores for GPT5 and Llama3.3 models evaluated in Polish and English.

Model	Lang	*n*	Run 1 Score	Run 2 Score
GPT5	PL	30	21.5 [16.0, 23.0]	21.3 [16.00, 23.00]
	EN	30	22.8 [16.0, 23.9]	22.06 [15.50, 23.56]
Llama3.3	PL	30	12.0 ± 5.9	11.51 ± 6.09
	EN	30	17.0 ± 4.6	17.30 ± 4.69

Note: Scores are total rubric scores on a 0–24 scale. Values are presented as Median [IQR] or Mean ± SD depending on normality.

## Data Availability

The original data presented in the study are openly available in DOI 10.5281/zenodo.19510134.

## Abbreviations

The following abbreviations are used in this manuscript:

AI-CDSS	AI-based Clinical Decision Support Systems
CCMUW	Clinical Center of Medical University of Warsaw
DDx	Differential Diagnoses
ICC	intraclass correlation coefficient
LEK	Lekarski Egzamin Końcowy/Polish Medical Final Examination
LLM	Large Language Model
MCQ	Multiple Choice Questions
PES	Państwowy Egzamin Specjalistyczny/State Specialization Exam

## Appendix A

### Appendix A.1

Example clinical case

**History of present illness** A 62-year-old man presented to his family doctor because of increasing sensation of shortness of breath on exertion. Over the past year, he had noticed that shortness of breath occurred when performing household chores and climbing stairs. Additionally, he experienced chronic fatigue. He didn’t report chest pain, dizziness or fainting. During the visit, he had a mild cold and reported a nonproductive cough.

**Past medical history** No significant chronic illnesses were identified. The patient has never been operated on. He does not smoke cigarettes and he does not consume alcohol. He has been on a vegan diet for years. In the past, he had taken iron supplements due to diagnosed microcytic anemia. Currently, he is taking no medications.

**Physical examination** Vitals: HR 88/min, regular; BP 128/73 mmHg; RR 17/min; T: 37.5 °C. General Appearance: the patient pale and in good general condition. Cardiovascular System: weak pulse on carotid arteries. On auscultation, a loud systolic murmur, most intense over the second intercostal space on the right side of the sternum; increased thoracic vibration, without displacement of apex beat. Respiratory System: normal vesicular murmurs, without additional sounds. Abdomen: soft, painless, peristalsis audible, without abnormalities. Musculoskeletal system: no abnormalities. Limbs/nervous system: no peripheral edema, neurological examination without abnormalities. Skin: pale.

**Laboratory** Results of complete blood count and biochemical tests within normal limits.

**Table A1:** Example scoring rubric.

Pool of Differential Diagnoses	Supporting Factors	Opposing Factors	Studies Required to Confirm the Diagnosis	Cost of the Studies (PLN)
Aortic stenosis	“increasing feeling of shortness of breath”; “shortness of breath occurs while performing household chores”; “weakly perceptible pulse in the carotid arteries” (small and sluggish pulse); “loud systolic murmur, most intense over the second intercostal space on the right side of the sternum”; “increased thoracic vibration”	“He didn’t report chest pain, dizziness or fainting”; “Blood pressure: 128/73 mmHg” (systolic pressure is within normal limits, although the range is not very narrow)	Transthoracic echocardiography (TTE)	280
Pericarditis	“T: 37.5 °C”; “he had a mild cold” (possible viral cause); “nonproductive cough”	“Over the past year, he had noticed that shortness of breath occurred” (pericarditis is usually acute and does not last a year); “a loud systolic murmur” (in pericarditis, there is pericardial friction rather than a systolic murmur); “He didn’t report chest pain” (pain is the primary symptom of pericarditis)	TTE + ECG	320
Microcytic anemia	“the patient pale”; “increasing sensation of shortness of breath”; “He has been on a vegan diet for years”; “he had taken iron supplements due to diagnosed microcytic anemia”	“Results of complete blood count and biochemical tests within normal limits” (excluding factor); “weak pulse on carotid arteries” (in anemia, blood circulation may be hyperkinetic, and the heart rate is usually high)	Complete blood count + Ferritin	62
Chronic coronary syndrome	“shortness of breath occurred when performing household chores and climbing stairs”; “increasing sensation of shortness of breath”; “A 62-year-old man” (risk factor)	“He didn’t report chest pain”; “He does not smoke cigarettes and he does not consume alcohol”; “He has been on a vegan diet for years” (lower risk of atherosclerosis); “Results of complete blood count and biochemical tests within normal limits”	TTE + ECG	320
Chronic heart failure	“increasing sensation of shortness of breath”; “shortness of breath occurred when performing household chores and climbing stairs”; “experienced chronic fatigue”; “a loud systolic murmur” (indicates an organic cause of heart failure)	“no peripheral edema” (no right ventricular failure symptoms); “normal vesicular murmurs, without additional sounds” (no pulmonary congestion at rest, which is possible during the stable phase or in HFpEF); “without displacement of apex beat”	TTE + BNP/NT-proBNP	405
Infective endocarditis	“T: 37.5 °C” (subfebrile state); “a loud systolic murmur” (possible valve damage); “experienced chronic fatigue”; “he had a mild cold”; “Skin: pale”	“Results of complete blood count and biochemical tests within normal limits” (no leukocytosis); “No significant chronic illnesses were identified” (no typical risk factors)	Blood cultures + TTE	316

### Appendix A.2

Prompts

**English:** You are an experienced internal medicine specialist solving a complex clinical case as part of a test. You will receive a case description. After reading it, present your answer in three parts.

Part One:

- List the three most probable diagnoses.
- For each of the three diagnoses, provide: (a) Supporting arguments—symptoms, test results, or risk factors that support this diagnosis. (b) Arguments opposing the diagnosis—symptoms or findings that contradict this diagnosis, or findings that would be expected but were not described. (c) Additional tests—which tests should be performed to confirm this diagnosis.

Part Two: Based on the reasoning above, provide one final diagnosis that you consider the most probable.

Part Three: State whether the patient in the described situation requires hospitalization. Answer unequivocally: YES or NO.

Answer always and exclusively in English.

**Polish:** Jesteś doświadczonym specjalistą chorób wewnętrznych, który rozwiązuje złożony przypadek kliniczny w ramach testu. Otrzymasz opis przypadku. Po jego przeczytaniu przedstaw odpowiedź w trzech częściach.

Część pierwsza:

- Wymień trzy najbardziej prawdopodobne diagnozy.
- Dla każdej z trzech diagnoz przedstaw: (a) Argumenty potwierdzające-objawy, wyniki badań lub czynniki ryzyka przemawiające za daną diagnozą. (b) Argumenty sprzeczne z diagnozą-objawy lub wyniki, które przeczą danej diagnozie, albo takie, które byłyby oczekiwane, lecz nie zostały opisane. (c) Badania dodatkowe-jakie badania należałoby wykonać, aby potwierdzić tę diagnozę.

Część druga:

- Na podstawie powyższego rozumowania podaj jedną ostateczną diagnozę, którą uważasz za najbardziej prawdopodobną.

Część trzecia:

- Odpowiedz, czy w opisanej sytuacji pacjent wymaga hospitalizacji. Odpowiedz jednoznacznie: TAK lub NIE.

Odpowiadaj zawsze i wyłącznie w języku polskim.
